# Extracorporeal membrane oxygenation (ECMO) assisted mediastinal tumor resection and superior vena cava replacement are safe and feasible

**DOI:** 10.1111/1759-7714.13140

**Published:** 2019-07-12

**Authors:** Shixin Zhang, Deli Tan, Wei Wu, Bo He, Tao Jing, Meng Tang, Tao Wu, Hongxiang Liu, Ming Zhang, Ni Zhou, Lingfeng Tang, Qiao Chen, Jinghua Tang, Mei Xia, Aihong Huang, Yi Liao, Yang Qiu, Haidong Wang

**Affiliations:** ^1^ Department of Thoracic Surgery, Southwest Hospital Army Medical University (Third Military Medical University) Chongqing China; ^2^ Department of Vasculocardiology, Southwest Hospital Army Medical University (Third Military Medical University) Chongqing China; ^3^ Department of Anesthesiology, Southwest Hospital Army Medical University (Third Military Medical University) Chongqing China; ^4^ Department of Cardiac Surgery, Southwest Hospital Army Medical University (Third Military Medical University) Chongqing China

**Keywords:** ECMO, mediastinal tumor resection, superior vena cava replacement

## Abstract

**Background:**

How to maximally improve the drainage of intracranial and upper body venous and to reduce neurological complications during thoracic tumor‐causedsuperior vena cava replacement are still clinical problems to be solved.

**Methods:**

We have innovatively used the bilateral jugular vein‐left femoral vein ECMO shunting to perform mediastinal tumor resection and superior vena cava replacement in a 50‐year‐old woman.

**Results:**

During the operation, this technique maintained the patient's hemodynamic stability, improved the cerebral oxygen saturation and reduced the cerebral ischemia, hypoxia as well as the neurological complications.

**Conclusion:**

It is indicated for patients with superior vena cava replacement who are unable to perform venous bypass (such as innominate vein to right atrial bypass) or venous shunting (such as differential pressure drainage from internal jugular vein to femoral vein).

## Introduction

Most patients with locally advanced lung cancer with superior vena cava invasion and invasive thymoma need to undergo superior vena cava resection and artificial vascular replacement during radical resection, which can improve their short‐ and long‐term survival rate.^1^, ^2^, ^3^ However, in the process of vascular replacement, the superior vena cava should be occluded. It causes a decrease in venous return and an increase in cerebral venous pressure which may result in hemodynamic instability and cerebral hypoperfusion, or even ischemia and hypoxia. Therefore, postoperative brain dysfunction occurs.^4^ If the superior vena cava is blocked, the superior vena cava blood flow can be improved to reduce cerebral venous pressure and neurological complications by venous bypass shunting, such as the innominate vein to the right atrial bypass or differential pressure drainage from the internal jugular vein to the femoral vein.^5^, ^6^, ^7^, ^8^ However, the innominate vein to right atrial bypass technique is not suitable for patients whose innominate vein is completely invaded by tumor. In addition, when performing the differential pressure drainage from the internal jugular vein to the femoral vein, because the venous pressure difference is not enough, some patients still have continuous elevated cerebral venous pressure. In surgery, it is required that the timespan from complete occlusion of the superior vena cava to the completion of the artificial vascular replacement is no more than 30 minutes, otherwise, irreversible brain damage may occur. Therefore, in the preoperative planning of such surgery, how to farthest improve the drainage of the intracranial and upper body veins and to reduce neurological complications remains a clinical problem to be solved. In this case, we used the internal jugular vein‐femoral vein ECMO (V‐V ECMO) bypass technique to perform mediastinal tumor resection and superior vena cava replacement. The mechanical centrifugal pump effectively implemented internal jugular vein‐femoral vein bypass, which solved the above problems well.

## Methods

**Figure 1:**
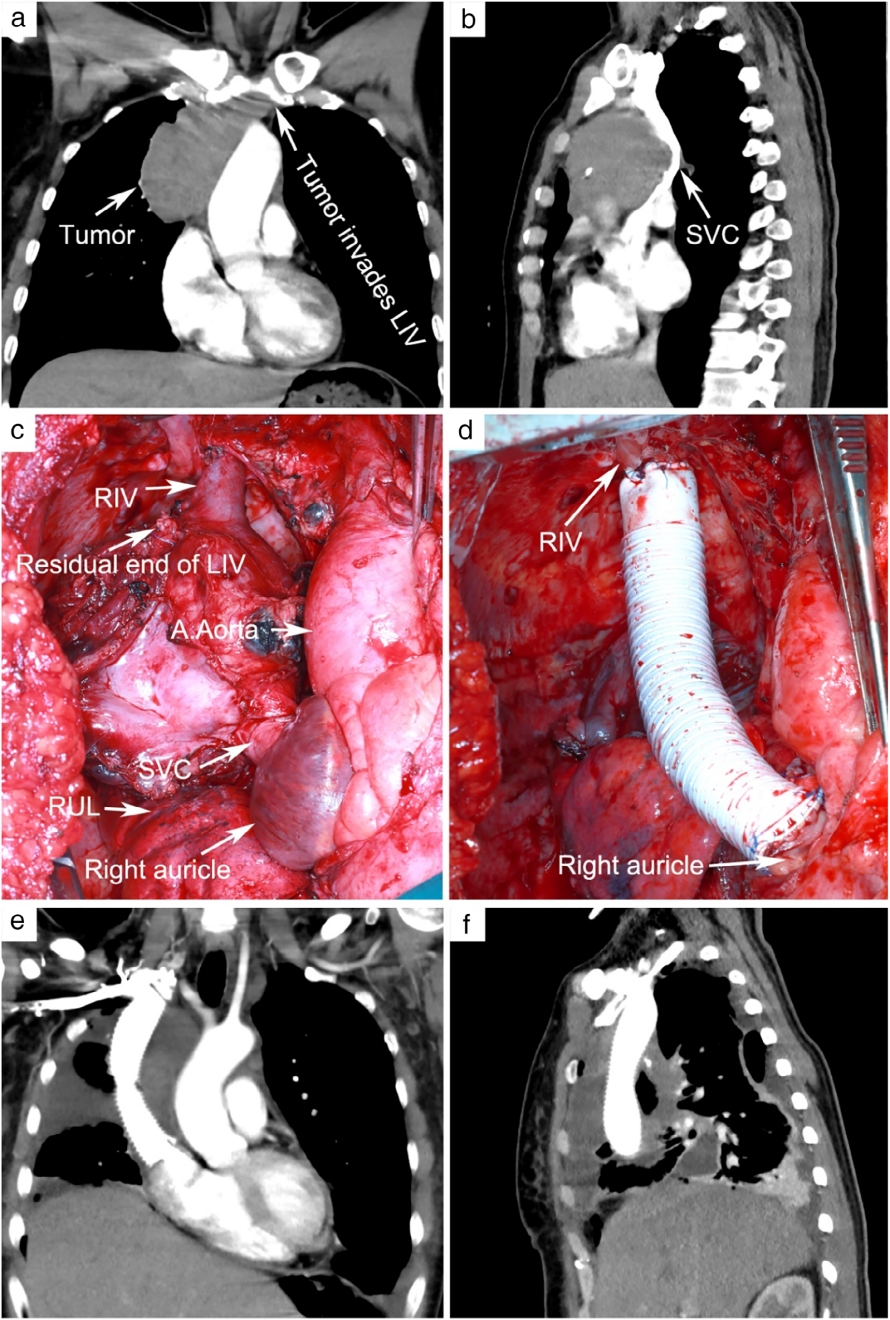
(**a** and **b**) Preoperative enhanced CT scan showed the tumor had invaded the left innominate vein and superior vena cava. Tumor thrombus was visible in the superior vena cava. (**c**) The tumor invaded most of the left innominate vein and the lower part of the right innominate vein to the superior vena cava 1 cm above the sinus node. (**d**) The artificial blood vessel was anastomosed to the right innominate vein and the right atrium. (**e** and **f**) Enhanced CT examination 10 days after surgery showed the unobstructed blood flow in the artificial vessel. LIV, left innominate vein; SVC, superior vena cava; RLV, right innominate vein; RUL, right upper lobe.

A 50‐year‐old female patient was admitted to our hospital in March 2019 with a history of cough, chest tightness with dizziness and headache for five months. Physical examination showed bilateral jugular vein engorgement, no edema in the face and upper limbs and the superficial vein of the chest and abdominal wall were not visible. Chest CT examination showed a soft tissue mass in the anterior middle and upper mediastinum. The size was approximately 6.7 cm × 7.0 cm. The tissue mass was closed to the superior vena cava which causing a severe stenosis of the lumen. Preoperative biopsy results were suggestive of thymoma. After the Preoperative multidisciplinary consultation and discussion, we thought: (i) The tumor invaded the left innominate vein and superior vena cava, and the tumor thrombus was observed in the superior vena cava which needed to be replaced (Fig 1a,b). (ii) If the uninvaded left innominate vein was long enough then a left innominate vein to right atrial bypass could be performed, otherwise an internal jugular vein to femoral vein shunt could be established. (iii) After shunting, if the patient had a decrease of blood pressure (systolic blood pressure <90 mmHg) and an increase of jugular venous pressure (CVP >40 mmHg), a V‐V ECMO bypass should be initiated to strengthen the shunt.

The operation was performed under combined intravenous anesthesia. A double lumen endotracheal tube was inserted in the left bronchus. The lateral internal jugular vein was punctured and a three‐channel tube and an 8.5 F vascular sheath tube placed, respectively, with their distal ends above the clavicles. The left femoral vein was punctured and an 8.5 F vascular sheath tube placed. During the operation, we used an ice cap for head cooling and continuously monitored the patient's electrocardiograph, arterial pressure, pulse oxygen saturation, bilateral internal jugular venous pressure, bilateral cerebral oxygen saturation and electroencephalogram. We recorded the above indicators at any three timepoints before blocking the superior vena cava, after occluding the superior vena cava and shunting in vitro, after initiating the ECMO shunting (flow rate: 0.8 L/min) and after loosening the artificial blood vessel. Each interval was at least five minutes, and blood was drawn from the left internal jugular vein at the third timepoint for blood gas analysis.

An incision was made crossing from the mid‐sternum to the fourth intercostal space of the anterolateral right chest. The main body of the tumor was located on the right side of the anterior superior mediastinum, invading the pericardium, the right upper lung, and most of the left innominate vein. Excision of the invaded pericardium and right upper lung revealed that most of the left innominate vein was invaded. Occlusion forceps were used to block the left innominate vein at a safe distance from the tumor, and a suture was made near the proximal end of the heart. The distal end above the occlusion forceps was not long enough for the anastomosis of the artificial blood vessel and it was therefore sutured. The tumor was then fully dissociated from the surrounding normal tissues, and only the superior vena cava, from the lower segment of the right innominate vein to 1 cm above the sinus node, was connected to the tumor, which could not be separated (Fig 1c). Heparin (0.5 mg/kg) was given by intravenous injection. The left and right internal jugular vein sheath tubes were connected in parallel and connected to the femoral vein sheath tube through an intravenous blood transfusion extension tube.

After blocking the superior vena cava by occlusion forceps, the patient's blood pressure, cerebral oxygen saturation continued to decrease and the CVP continued to rise. Electroencephalogram indicated cerebral ischemia and hypoxia, and the occlusion forceps were subsequently released to ensure venous return. After the ECMO pipes were prefilled, the front end of the pump was connected to the bilateral internal jugular vein sheath tube through the blood transfusion extension tube, and the posterior end of the membranous lung was connected to the femoral vein sheath tube through the blood transfusion extension tube. Then, V‐V ECMO flow was initiated, and the flow was gradually increased to 0.8 L/min. The right innominate vein was blocked close to the chest wall and cutoff 0.5 cm above the occlusion forceps. Meanwhile, the superior vena cava was disconnected 0.5 cm above the sinus node, and sutured near the proximal end of the heart. The GORE‐TEX ring artificial blood vessel was trimmed to the appropriate length and soaked in heparin water. The artificial blood vessel and the right innominate vein end were continuously eversion sutured with a 4–0 Prolene line, and the proximal end of the artificial blood vessel was anastomosed to the right atrium end (Fig 1d). After anastomosis, artificial vascular exhaust was performed. The occlusion forceps were then released, and the ECMO flow was stopped followed by removal of the tubes and sheaths. Heparin was neutralized by protamine after monitoring the activated clotting time (ACT).

## Results

**Figure 2:**
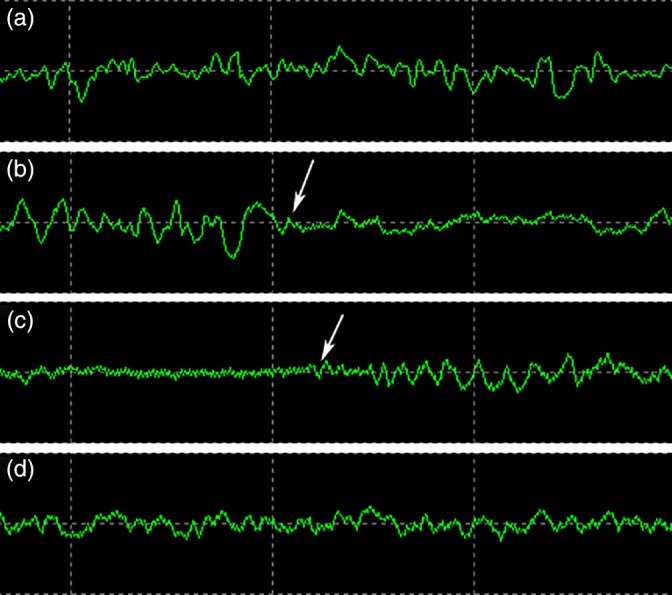
(**a**) EEG amplitude was normal before blocking the superior vena cava. (**b**) After blocking the superior vena cava, the EEG amplitude was reduced (shown by the white arrow). (**c**) After ECMO shunting was switched on, the EEG amplitude increased significantly (shown by the white arrow). (**d**) After opening the artificial blood vessel, the EEG amplitude returned to normal. EEG, electroencephalogram.

Intraoperatively, the patient's heart rate ranged from 48 to 110 beats per minute, all of which were sinus rhythm. Before blocking the superior vena vein, except for the pressure in the left and right jugular veins was 19 ± 2.1 and 16 ± 1.2 mmHg, respectively, the other indicators were normal. After blocking the superior vena cava and shunting in vitro, the pressure in the left and right jugular veins was 47 ± 7.0 and 42 ± 8.6 mmHg, respectively, the arterial systolic pressure decreased to 73 ± 6.2 mmHg, and the average pressure was 59 ± 5.3 mmHg. The mixed blood oxygen saturation of the left and right brain was 51 ± 5.4% and 47 ± 5.7%, respectively. Then, after initiating the ECMO shunting (flow rate: 0.8 L/min), the pressure in the left and right jugular veins was 22 ± 2.5 and 20 ± 16 mmHg, the arterial systolic pressure was 92 ± 2.9 mmHg, the average pressure was 69 ± 2.6 mmHg, and the mixed blood oxygen saturation of the left and right brains was 62 ± 1.2% and 61 ± 1.2%, respectively. When the artificial blood vessel was opened, all indicators returned to normal. Before blocking the superior vena cava, the electroencephalogram (EEG) amplitude was normal (Fig 2a), while after blocking, the EEG amplitude was reduced suggesting cerebral ischemia and hypoxia (Fig 2b). After ECMO running, the EEG amplitude increased significantly suggesting a significant improvement in cerebral ischemia and hypoxia (Fig 2c). When the artificial blood vessel was opened, the EEG amplitude returned to normal (Fig 2d). Intraoperative pulse oxygen saturation fluctuated between 91% and 100%. The blood lactic acid was normal following blood gas analysis of the left internal jugular vein. The time of blocking the superior vena cava was 65 minutes, and the ECMO running was 75 minutes. Ten minutes after ECMO running, the ACT was 243 seconds, and 10 minutes after the ECMO switched off, the ACT was 185 seconds.

After surgery, the patient returned to the thoracic surgery intensive care unit with a tracheal catheter in situ. A total of 250 mL of 20% human blood albumin, 40 mg of methylprednisolone and 250 mL of glycerine‐fructose were intravenously injected. Eight hours after surgery, the patient was called to open her eyes. The tracheal catheter was removed, and the patient resumed spontaneous breathing. The left upper limb was slightly swollen without neurological complications. Therefore, 250 mL of glycerine‐fructose was intravenously injected for dehydration, and furosemide was given for diuresis. On the second day after the surgery, low‐molecular‐weight heparin and warfarin were administered for anticoagulation. On the third day after the bridging, rechecking the PT was 25 seconds and INR was 2.2. Therefore, heparin was discontinued and warfarin was given alone for anticoagulant therapy. On the third day after the surgery, the swelling of the patient's left upper extremity subsided. The patient was discharged 10 days after surgery. Before discharge, chest enhanced CT examination showed unobstructed blood flow in the artificial vessel (Fig 1e,f). Postoperative pathology results were suggestive of thymoma (type B1).

## Discussion

In patients with lung cancer or mediastinal tumor invading the superior vena cava, if there is no surgical contraindication, the superior vena cava should be formed or replaced while the tumor is being resected. In surgery, after the superior vena cava is blocked, the returned blood volume reduces causing a sudden drop in blood pressure which can induce arrhythmia and/or cardiac arrest. More importantly, it will result in an increase in cerebral venous pressure and a decrease in cerebral blood flow, or even a cessation of circulation, causing damage to the central nervous system.^4^, ^9^ Therefore, maintaining the stability of the circulatory system and ensuring effective cerebral blood flow and alleviating ischemic damage of the brain are the keys to treatment during this period. Traditional brain protection measures include ice cap cooling, reduction of blood pressure before vascular occlusion, minimizing the blocking time and giving dehydration treatment such as furosemide and mannitol to relieve brain edema. However, these measures have poor controllability and no significant relief of the superior vena cava pressure.

Currently, the conventional method to replace the superior vena cava is partial blocking of the superior vena cava for direct repair or establishing the left innominate vein‐right atrial bypass before blocking the superior vena cava.^10^ However, this method is not suitable for cases in which the left innominate vein is completely invaded by tumor. Meanwhile, it also affects the surgical field of view and increases the operation time.^9^, ^11^ Another alternative method is inserting the Swan‐Ganz sheath tube in the jugular and femoral vein, respectively. The two are connected through a blood transfusion extension tube to perform extracorporeal differential pressure drainage.^12^ In order to maintain circulation, the dosage of vasoconstrictive drugs such as norepinephrine should be increased during this surgical procedure. However, even if the circulation is maintained stable, some patients still experience a sharp increase in jugular vein pressure. Moreover, it is impossible to accurately monitor the volume of drainage during the operation. In addition, extracorporeal circulation for superior vena cava replacement has also been reported.^13^ This method is more widely used in patients with simultaneous invasion of atrium and/or aorta by tumors.^14^ All of the above techniques have been shown to maintain hemodynamic stability, reduce intracranial pressure and reduce neurological complications, but they all have certain limitations, which required us to carefully choose the surgical plan according to the patient's condition.

In this case, preoperative CT examination revealed that the tumor was located in the right anterior superior mediastinum and invaded the left innominate vein. Therefore, the jugular vein and femoral vein sheath were prereserved for extracorporeal diversion. Intraoperative exploration confirmed that the tumor almost invaded the full length of the left innominate vein, indicating that it was not suitable to perform the intrathoracic left innominate vein‐right atrial shunt. When performing the left and right innominate vein‐femoral differential pressure drainage after blocking the superior vena cava, the patient experienced a decrease in blood pressure, an increase in jugular venous pressure, a decrease in cerebral oxygen saturation, and changes of cerebral ischemia and hypoxia. We speculated that it was related to insufficient drainage of venous blood from the upper to the lower body. To solve this problem, we innovatively used V‐V ECMO technology by connecting the bilateral internal jugular vein with the pipe of the ECMO pump via the sheath tubes and an intravenous blood transfusion extension tube. By ECMO shunting, oxygenated blood at the posterior end of the membranous lung was pumped back to the femoral vein through an intravenous blood transfusion extension tube and the sheath tube. We deliberately cut the ECMO tube and only keep an appropriate length to reduce the blood volume and blood flow resistance of the extracorporeal tube. When the ECMO flow rate was 0.8 L/min, the above indicators were significantly improved, suggesting that the upper body venous blood could be fully drained to the femoral vein through V‐V ECMO bypass.

We believe that the value of using ECMO in the thoracic tumor treatment required superior vena cava replacement for the following reasons. (i) It effectively solves the problem of upper body venous blood return, so that the operation time of blocking the superior vena cava can be significantly more than 30 minutes, which can provide a safety guarantee for special patients with more complicated conditions. (ii) The ECMO pump can actively ensure the effective flow, which can be dynamically adjusted according to internal jugular vein pressure, cerebral oxygen saturation, electrophysiology and other objective indicators; (iii) Under close monitoring, it is safe, efficient and suitable for all thoracic tumors necessitating superior vena cava replacement.

In conclusion, in the process of occlusion of the superior vena cava and replacement with the artificial vessel, we innovatively used the bilateral jugular vein‐left femoral vein ECMO shunting to actively and effectively reduce the bilateral internal jugular venous pressure, to maintain hemodynamic stability, improve cerebral oxygen saturation and reduce cerebral ischemia, hypoxia and neurological complications. We believe that our method is safe, effective and feasible if appropriate patients are selected in clinical practice.

## Disclosure

No authors report any conflict of interest.

## References

[1] Ciccone AM , Venuta F , D'Andrilli A *et al* Long‐term patency of the stapled bovine pericardial conduit for replacement of the superior vena cava. Eur J Cardiothorac Surg 2011; 40: 1487–91. 21530293 10.1016/j.ejcts.2011.03.008

[2] Shargall Y , de Perrot M , Keshavjee S *et al* 15 years single center experience with surgical resection of the superior vena cava for non‐small cell lung cancer. Lung Cancer 2004; 45: 357–63. 15301876 10.1016/j.lungcan.2004.02.009

[3] Spaggiari L , Leo F , Veronesi G *et al* Superior vena cava resection for lung and mediastinal malignancies: A single‐center experience with 70 cases. Ann Thorac Surg 2007; 83: 223–9. 17184668 10.1016/j.athoracsur.2006.07.075

[4] Leo F , Della Grazia L , Tullii M *et al* Hemodynamic instability during superior vena cava crossclamping: Predictors, management, and clinical consequences. J Thorac Cardiovasc Surg 2007; 133: 1105–6. 17382668 10.1016/j.jtcvs.2006.11.020

[5] Dai W , Dong J , Zhang H , Yang X , Li Q . Superior vena cava replacement combined with venovenous shunt for lung cancer and thymoma: A case series. J Thorac Dis 2018; 10: 363–70. 29600068 10.21037/jtd.2017.12.130 PMC5863137

[6] Sekine Y , Suzuki H , Saitoh Y , Wada H , Yoshida S . Prosthetic reconstruction of the superior vena cava for malignant disease: Surgical techniques and outcomes. Ann Thorac Surg 2010; 90: 223–8. 20609780 10.1016/j.athoracsur.2010.03.050

[7] Spaggiari L , Thomas P , Magdeleinat P *et al* Superior vena cava resection with prosthetic replacement for non‐small cell lung cancer: Long‐term results of a multicentric study. Eur J Cardiothorac Surg 2002; 21: 1080–6. 12048089 10.1016/s1010-7940(02)00175-6

[8] Sun Y , Gu C , Shi J *et al* Reconstruction of mediastinal vessels for invasive thymoma: A retrospective analysis of 25 cases. J Thorac Dis 2017; 9: 725–33. 28449480 10.21037/jtd.2017.03.03 PMC5394000

[9] Galatoudis Z , Soumpasis I , Vretzakis G . Anesthetic considerations for surgery involving clamping of superior vena cava. Greek E‐J Perioper Med 2005; 3: 49–59.

[10] Garcia A , Flores RM . Surgical management of tumors invading the superior vena cava. Ann Thorac Surg 2008; 85: 2144–6. 18498851 10.1016/j.athoracsur.2007.12.027

[11] Nazari S , Moncalvo F , Zonta A , Prati U , Jemos V . Temporary intraluminal bypass for superior vena cava reconstruction after cancer invasion. Thorac Cardiovasc Surg 1988; 36: 5–9. 3376089 10.1055/s-2007-1020033

[12] Perentes JY , Erling CC , Ris HB , Corpataux JM , Magnusson L . A simple bypass technique for superior vena cava reconstruction. Interact Cardiovasc Thorac Surg 2011; 12: 15–9. 20937665 10.1510/icvts.2010.247205

[13] Byrne JG , Leacche M , Agnihotri AK *et al* The use of cardiopulmonary bypass during resection of locally advanced thoracic malignancies: A 10‐year two‐center experience. Chest 2004; 125: 1581–6. 15078778 10.1378/chest.125.4.1581

[14] Ried M , Neu R , Schalke B , von Süßkind‐Schwendi M , Sziklavari Z , Hofmann HS . Radical surgical resection of advanced thymoma and thymic carcinoma infiltrating the heart or great vessels with cardiopulmonary bypass support. J Cardiothorac Surg 2015; 10: 137. 26515387 10.1186/s13019-015-0346-2 PMC4627626

